# Factors influencing the quality of life of pregnant women: a systematic review

**DOI:** 10.1186/s12884-018-2087-4

**Published:** 2018-11-23

**Authors:** Nolwenn Lagadec, Magali Steinecker, Amar Kapassi, Anne Marie Magnier, Julie Chastang, Sarah Robert, Nadia Gaouaou, Gladys Ibanez

**Affiliations:** 1 Department of Education and Research in General Medicine, Faculty of Medicine Pierre et Marie Curie, 27, rue Chaligny – 75571, cedex 12 Paris, France; 2 Sorbonne Universités, UPMC Univ Paris 06, INSERM, Institut Pierre Louis d’épidémiologie et de Santé Publique (IPLESP UMRS 1136), F75012 Paris, France

**Keywords:** Quality of life, Pregnancy, Primary care, Health-related quality of life, Measurement

## Abstract

**Background:**

Pregnancy is a period of transition with important physical and emotional changes. Even in uncomplicated pregnancies, these changes can affect the quality of life (QOL) of pregnant women, affecting both maternal and infant health. The objectives of this study were to describe the quality of life during uncomplicated pregnancy and to assess its associated socio-demographic, physical and psychological factors in developed countries.

**Methods:**

A systematic review was performed according to the PRISMA guidelines. Searches were made in PubMed, EMBASE and BDSP (Public Health Database). Two independent reviewers extracted the data. Countries with a human development index over 0.7 were selected. The quality of the articles was evaluated on the basis of the STROBE criteria.

**Results:**

In total, thirty-seven articles were included. While the physical component of QOL decreased throughout pregnancy, the mental component was stable and even showed an improvement during pregnancy. Main factors associated with better QOL were mean maternal age, primiparity, early gestational age, the absence of social and economic problems, having family and friends, doing physical exercise, feeling happiness at being pregnant and being optimistic. Main factors associated with poorer QOL were medically assisted reproduction, complications before or during pregnancy, obesity, nausea and vomiting, epigastralgia, back pain, smoking during the months prior to conception, a history of alcohol dependence, sleep difficulties, stress, anxiety, depression during pregnancy and sexual or domestic violence.

**Conclusions:**

Health-related quality of life refers to the subjective assessment of patients regarding the physical, mental and social dimensions of well-being. Improving the quality of life of pregnant women requires better identification of their difficulties and guidance which offers assistance whenever possible.

## Background

According to the World Health Organization, quality of life (QOL) is defined as “*individuals’ perception of their position in life in the context of the culture and value systems in which they live and in relation to their goals, expectations, standards and concerns. This is a very broad concept, and one that can be influenced in a complex way by the physical health of the subject, his or her psychological state and level of independence, social relations and relationship with the essential elements of his or her environment*” [1]. It is therefore founded on several objective factors (linked to the quality of the environment and living conditions), and subjective factors (linked to the personal sphere and measurable in terms of satisfaction and well-being). Health status as an essential component of quality of life is referred to as health-related quality of life (HRQOL) [2].

Pregnancy is a period of transition with important physical and emotional changes [3]. Even in uncomplicated pregnancies, these changes can affect the quality of life of pregnant women and affect both maternal and infant health (pregnancy monitoring, pregnancy outcomes, maternal postpartum health, and the psychomotor development of the infant) [4–8]. Health professionals in the field of prenatal maternal and child health try to satisfy their patients with respect to their experience during preconception and pregnancy periods [2]. Traditionally used pregnancy outcome measures, such as morbidity and mortality rates, remain essential. However, they are not sufficient on their own because population health should be assessed, not only on the basis of saving lives, but also in terms of improving quality of life [2, 9].

Over the past decades, numerous instruments have been developed to measure HRQOL in various patient populations, with 2 basic approaches: generic and disease-specific [10]. While generic measures (for example the SF-36 Short-Form Item 36 and WHOQOL-BREF World Health Organization’s Quality of Life Scale) have broad application across different types and severity of diseases, disease-specific measures are designed to assess particular diseases or patient populations. To our knowledge, there is no review of the literature to describe the quality of life of pregnant women in primary care. The objectives of this study were to describe the quality of life during uncomplicated pregnancy and to assess its associated socio-demographic, physical and psychological factors in developed countries.

## Methods

### Type of study

The study consisted of a systematic review of the literature in the PUBMED, EMBASE and BDSP databases (BDSP is the French Public Health Database). The search was performed according to the Preferred Reporting Items for Systematic Reviews and Meta-Analyses (PRISMA) criteria.

### Inclusion and exclusion criteria

The search strategy and inclusion/exclusion criteria were developed by the whole group of authors after which two authors (LN and IG) individually conducted the literature search. Key search terms included “Pregnancy”, “Quality of life”, or “Health related quality of life”. Search terms were selected with reference to relevant index terms (MeSH, Emtree or Thesaurus). All observational studies (e.g., cohort, cross-sectional, case-control) which were published in English and French prior to March 2016 have been considered (no restriction in the starting date). Developed countries were chosen as a basis for the research to ensure epidemiological uniformity. In order to define a list of developed countries comparable to France, countries with a human development index (HDI) of over 0.7 were selected. This list, provided by the United Nations (UN), is available online [11]. Studies measuring quality of life with a single question were excluded [12, 13]. Studies on specific populations (women with complicated pregnancy) or on a specific scale of quality of life (sexual HRQOL or HRQOL in relation to faecal incontinence, etc.) were also excluded, as it is not possible to compare patients with different pathologies.

### HRQOL measurement

The most frequently used HRQOL instruments during pregnancy are the Medical Outcomes Study Short Form 36 survey (SF-36), the Medical Outcomes Study Short Form 12 survey (SF-12), the World Health Organization’s Quality of Life Scale (WHOQOL) and the World Health Organization’s Quality of Life Scale – BREF (WHOQOL – Bref).

The SF-36 includes 36 items and collects information on eight health concepts including, physical functioning, role limitations due to physical and emotional health, mental health, bodily pain, general health, vitality and social functioning. These items are scored providing a component summary scale score for both mental (SF36-MCS) and physical (SF36-PCS) HRQOL (from 0 to 100) [14]. The SF-12 is a validated shortened version of the SF-36. A lower score on the summary scales represents a poorer HRQOL.

The WHOQOL includes 100 questions grouped into 6 categories (physical, psychological, independence, social, environmental and spiritual) (from 0 to 100). The WHOQOL-BREF instrument comprises 26 items and is a validated shortened version of the WHOQOL. A lower score on the summary scales represents a poorer HRQOL.

### Article selection and quality assessment

A preliminary selection was made from the titles, then another on reading the summaries and a lastly on a reading of the entire article. Publications “related” to the selected articles as well as the bibliography of the selected articles were also examined. Two independent reviewers extracted the data (LN and IG). Disagreement between reviewers was resolved by consensus. The quality of the articles was evaluated using the STROBE criteria (STrengthening the Reporting of OBservational studies in Epidemiology) [15].

### Analysis

The results were organised in two sections: first, a description of the quality of life of pregnant women in developed countries and second, by the socio-demographic, physical and psychological factors associated with their quality of life. When the study compared two quality of life scales, we used only the “Gold Standard” scale. In the case-control studies where the controls were a particular subgroup, we have retained the “control” group that was most representative of the general population of pregnant women, where all subjects in the study would have resulted in a serious selection bias.

## Results

### Article selection

**Fig. 1:**
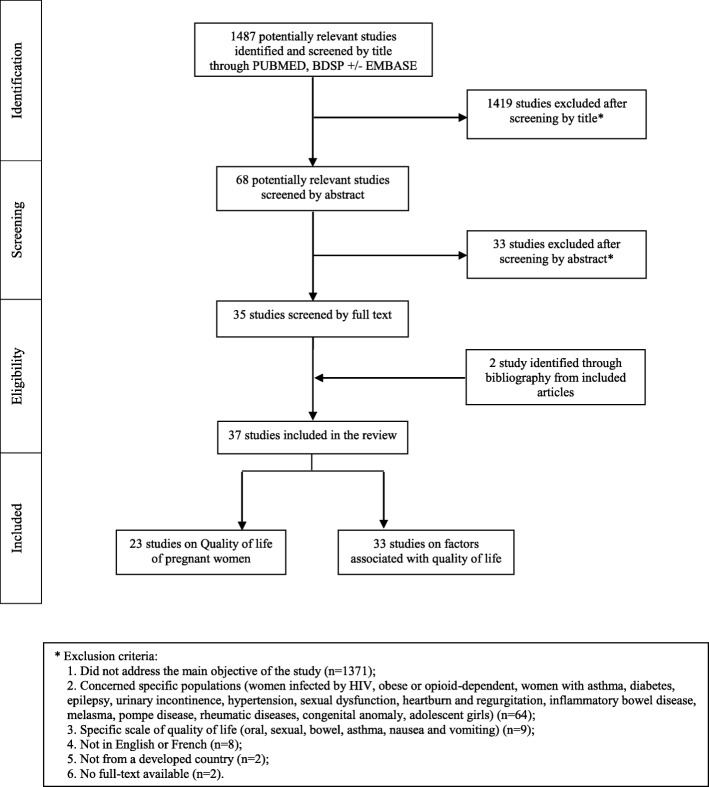
Flow chart of the study selection

The article selection is described in Fig. 1. Of the 1487 articles retrieved, 37 were selected for our analysis (Fig. 1 and Table 1). The methodological quality was rated from 11 to 22 in the selected articles. The selected articles were published between 2001 and 2016. The samples of pregnant women included in the studies varied between 55 and 12,056 women. Concerning the design of the selected studies, twenty were cross-sectional studies [16–35], four were case-controlled studies [36–38], and fourteen were longitudinal cohort studies [14, 39–51]. Thirteen studies were conducted during the first trimester of pregnancy [17, 20, 22, 23, 26, 30, 38–40, 44–47], eleven were from the second trimester [14, 16, 24, 35, 38–41, 45, 46, 48], eighteen were from the 3rd trimester [14, 18, 19, 27, 29, 31, 33, 34, 37–42, 46, 48–50] and six studies focused on the entire pregnancy [25, 28, 32, 36, 43, 51]. In measuring the quality of life, nineteen studies used SF-36 [14, 17–19, 21, 22, 25–27, 30, 33, 34, 38–41, 44, 46, 51], twelve studies used SF-12 [16, 20, 23, 24, 28, 29, 35, 42, 43, 46, 48, 50], two studies used the WHOQOL Brief [32, 47], one study used The Duke Health Profile [49], and another Nottingham Health Profile [31].

**Table 1:** Studies characteristics

Author,Year, Reference	Study design	Number of participants	Response rate	QOL scale used	STROBE rate
Abbasi M. et al.(2013) Iran [38]^a^	Prospective cohort study	1550	66%	Medical Outcomes Study Short Form 36 (SF-36)	20
Aquino NM (2009) Brasil [15]^b^	Cross-sectional study	179	99,40%	Standard Short Form 12 Health Survey (SF-12)	18
Chan OK et al. (2010) China [16]^a,b^	Cross-sectional study	418	94,70%	Medical Outcomes Study Short Form 36 (SF-36)	17
Chang et al. (2014) Taiwan [40] ^a,b^	Longitudinal cohort study	410	83,30%	Medical Outcomes Study Short Form 36 (SF-36)	19
Coban A. et al. (2011) Turkey [36]^b^	Case-controlled study	100	–	WHO-QOL-BREF Questionnaire	13
Da Costa D et al. (2010) Canada [18] ^a,b^	Cross-sectional study	245	66,60%	Medical Outcomes Study Short Form 36 (SF-36)	17
Dall’Alba V et al. (2015) Brasil [18]^b^	Cross-sectional study	82	–	Medical Outcomes Study Short Form 36 (SF-36)	13
De Pascalis L*.* et al. (2012) Italy [40]^a,b^	Case-controlled study	115	87,79	Medical Outcomes Study Short Form 36 (SF-36)	19
Elsenbruch S. et al. (2006) Germany [19]^a,b^	Cross-sectional study	978	91,60%	Medical Outcomes Study, Short Form 12 (SF-12)	19
Emmanuel EN et al. (2012) Australia [50]^a, b^	Longitudinal cohort study	630	77%	Medical Outcomes Study Short Form 12 (SF-12) Version 2	17
Emmanuel EN et Sun J. (2014) Australia [42] ^a,b^	Longitudinal cohort study	605	60%	Medical Outcomes Study, Short Form 12 (SF-12)	18
Fatemeh A et al. (2010) Iran [32]^a,b^	Cross-sectional study	600	–	Medical Outcomes Study Short Form 36 (SF-36)	12
Gezginç K et al., (2008) Turkey [35]^b^	Cross-sectional study	55	–	WHO-QOL-BREF Questionnaire	14
Gharacheh M. et al., (2015) Iran [20]^b^	Cross-sectional study	328	96%	Medical Outcomes Study Short Form 36 (SF-36)	19
Haas JS et al. (2004) USA [42]^a, b^	Longitudinal cohort study	2854	63%	Medical Outcome Study Short Form-36 (SF-36)	20
Hama K et al. (2008) Japan [51]^b^	Longitudinal cohort study	190	83%	Medical Outcomes Study Short Form 36 (SF-36) version 2	14
Jomeen J., Martin CR; (2005) United Kingdom [21]^a,b^	Cross-sectional study	129	–	Medical Outcomes Study Short Form 36 (SF-36) Version 2	15
Lacasse A et al. (2008) Canada [23] ^a,b^	Cross-sectional study	507	77%	Medical Outcomes Study, Short Form 12 (SF-12)	18
Lau Y et al. (2011) China [23]^b^	Cross-sectional study	1151	71,4%	Standard Short Form 12 Health Survey (SF-12)	18
Li J et al. (2012) China [24]^b^	Cross-sectional study	454	79%	Medical Outcomes Study Short Form 36 (SF-36) version 2	17
Liu L et al.(2013) USA [25]^b^	Cross-sectional study	195	88%	Medical Outcomes Study Short Form 36 (SF-36)	19
Mckee MD et al (2001) USA [26]^b^	Cross-sectional study	114	74%	Medical Outcomes Study Short Form 36 (SF-36)	18
Mota N. et al. (2008) Canada [27]^a^	Cross-sectional study	12,056	81,20%	Medical Outcomes Study, Short Form 12 (SF-12)	19
Moyer CA et al. (2009) USA, China, Ghana [29] ^a,b^	Cross-sectional study	251 Chine, 311 USA	–	Medical Outcomes Study, Short Form 12 (SF-12)	14
Nakamura Y. et al. (2012) Japan [38] ^a,b^	Case-controlled study	692	66,10%	Medical Outcomes Study Short Form 36 (SF-36) Version 2	16
Ngai FW, Ngu SF. (2013) Hong Kong [43] ^a^	Prospective cohort study	256	79,3%	Medical Outcomes Study, Short Form 12 (SF-12)	17
Nicholson WK (2006) USA [29]^b^	Cross-sectional study	221	79%	Medical Outcomes Study Short Form 36 (SF-36)	19
Olsson C, Nilsson-Wikmar L.(2004) Sweden [31] ^b^	Cross-sectional study	136	85%	Nottingham Health Profile	22
Ramirez-Vélez R (2011) Colombia [34]^a, b^	Cross-sectional study	64	–	Medical Outcomes Study, Short Form 12 version 2 (SF-12 V2)	17
Setse R et al. (2008) USA [44]^b^	Prospective cohort study	200	81%	Medical Outcomes Study Short Form 36 (SF-36)	20
Shishehgar S*.* et al. (2014) Iran [31]^a, b^	Cross-sectional study	210	–	WHO-QOL-BREF Questionnaire	15
Tavoli Z et al. (2016) Iran [33]^a, b^	Cross-sectional study	266	86,5%	Medical Outcomes Study Short Form 36 (SF-36)	19
Tendais I et al. (2011) Portugal [45]^b^	Prospective cohort study	56	56%	Medical Outcomes Study Short Form 36 (SF-36)	16
Tsai SY et al. (2016) Taiwan [46]^a, b^	Prospective cohort study	172	95,3%	Medical Outcomes Study, Short Form 12 version 2 (SF-12 V2)	18
Vachkova E et al. (2013) Czech Republic [47]^a^	Prospective cohort study	225	90,60%	WHO-QOL-BREF Questionnaire	11
Vinturache A. et al. (2015) Canada [48]^a, b^	Prospective cohort study	3388	99%	Medical Outcomes Study, Short Form 12 (SF-12)	18
Wang P et al.(2013) Taiwan [49]^a, b^	Prospective cohort study	265	78,86%	Duke Health Profile (DUKE)	20

List of abbreviations: *QOL* Quality of life, ^a^First research question: quality of life of pregnant women in developed countries; ^b^second research question: factors associated with their quality of life

### The quality of life of pregnant women

#### Comparison with the general population

The quality of life of pregnant women was generally lower than that of the general population. Two studies explicitly compared their results with those of non-pregnant women of the same age. On the SF-36 scale, Da Costa et al. found physical activity and physical pain values equal to 56.7 and 61.7; these values were 90.9 and 75.0 for non-pregnant Canadian women of the same age [18]. Similarly, Nakamura et al. made similar comparisons in Japan [38]. The Chan et al. study in 2010 also found that pregnant women had, on average, statistically lower QOL scores (*p* < 0.001) compared to the general population, excepting general health (*p* = 0.1) [17, 18]. Similarly, Elsenbruch et al. [20] found a reduced quality of life, physically, when compared to German women of the same age (*p* < 0.001).

#### The progression of the quality of life during the trimesters

**Fig. 2:**
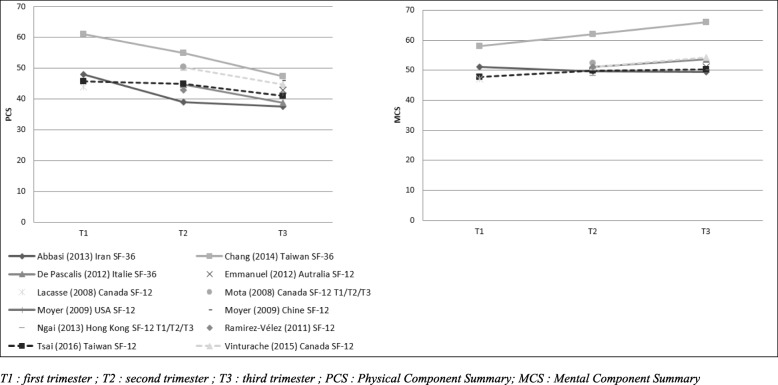
Changes in PCS and MCS over trimesters

**Fig. 3:**
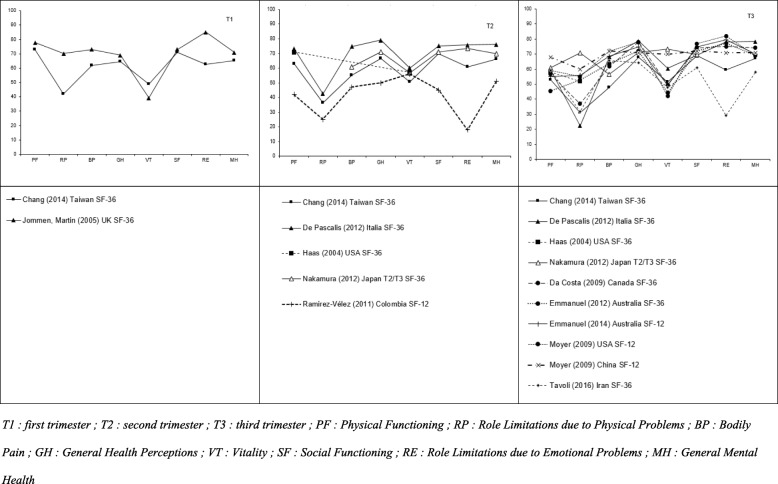
Evolution of the 8 dimensions of SF-36 during the trimesters

Of the 23 studies selected, 20 (86,9%) described the progression of QOL using SF-36 or SF-12 (Figs. 2, 3). The study of the SF-36 PCS and MCS aggregate scores revealed that there were significant variations during the trimesters (Fig. 2).

The PCS values ranged from 48 to 61 during the 1st trimester; between 39 and 55 during the 2nd trimester; between 37.5, and 47.5 during the 3rd trimester. For SF-12, values ranged from 44 to 46 in the 1st trimester, 43 and 50 in the 2nd trimester and 41 and 45 in the 3rd trimester. The results of the studies indicated a decrease in physical quality of life throughout pregnancy, particularly related to decreased physical activity and functional limitations (related to physical health and physical pain). In terms of prevalence, the Haas et al. study in 2005 showed an increase in pregnant women with poor physical quality of life during pregnancy: 9% of pregnant women in the second trimester, and 13% in the third trimester [14]. The proportion of pregnant women reporting generally poor health (score 0 to 50) increased from 15.5 to 20.1 and 26.9% and then decreased to 21% in the postpartum period [49].

The MCS values were as follows for the SF-36: in the 1st trimester values between a minimum of 51 and a maximum of 58; in the 2nd trimester between 49 and 62; in the 3rd trimester between 49.5 and 66. In parallel with SF-12, the MCS was between 47 and 48 in the first trimester, between 49 and 52 in the second trimester and 50 and 54 in the third trimester. In five studies, the quality of mental life of the pregnant women increased or remained stable over the course of the trimesters (Fig. 2).

The evolution of the 8 dimensions of SF-36 during the quarters is presented in Fig. 3. The following domains had the lowest scores: role limitations due to physical problems (RP) and vitality (VT). The following domains had the highest scores: general health perceptions (GH) and bodily pain (BP). The results concerning the role limitations due to emotional problems (RE) varied across studies.

### Factors influencing quality of life

The results of studies on the factors associated with the quality of life of pregnant women are presented in Tables 2 and 3. Thirty-three articles were selected for this section.

**Table 2:** Factors significantly associated with poor quality of life according to the studies (*p* < 0.05)

Risk factors	Protective Factors
Socio-demographic characteristics	
Older pregnant women [18, 23, 25, 42]	
Economic difficulties [14, 30, 32, 35]	
Low level of education [18, 25, 33, 35, 49]	
Unemployment [25, 49]	Occupation (housewife) [35]
Ethnic minority [30]	Ethnic minority [23, 42]
Isolation	
Single [18, 35, 42]	Single [25]
No partner support [18]	
No social support [18, 20, 30]	
Little / no partner support [18]	
Medical characteristics	
Adverse medical history [18]	
Obesity [14, 48]	Obesity [25]
Bad physical condition prior to conception [43, 49]	
Smoking prior to conception [14]	
Addiction to alcohol prior to conception [14]	
Practicing physical exercise [14, 23, 45]	
Obstetrical characteristics	
Experience of infertility [16]	
Primiparity [48, 51]	Primiparity [18, 33, 40, 49]
More advanced in weeks pregnant [16, 28, 33]	More advanced in weeks pregnant [39-mcs]
Hospitalisation during pregnancy [25]	
Medically assisted reproduction [14, 40, 41]	Medically assisted reproduction [48]
Obstetric Complications [18, 21, 40]	
Psychological characteristics	
Prenatal depressive symptoms [18, 22, 25, 30, 44]	
Stress, prenatal anxiety [18, 22, 24, 32, 42]	
History of sexual violence [16]	
History of domestic violence [21, 34]	
Difficult life events [18]	
	Wanted pregnancy [25, 33, 40]
	Happy being pregnant [49]
	Optimistic [29] Life Satisfaction [33]
Symptoms during pregnancy	
Nausea / vomiting [14, 17, 23]	
Epigastralgia / reflux [19]	
Back pain [14, 31]	
Sleep disorder [14, 18, 46]	
Comfort [38]

**Table 3:** Factors Associated with Quality of Life During Pregnancy

Author, Year, Country	Factors related to QOL *	Key Results
Aquino NM (2009) [17]	Sexual Violence *	Women who had experienced sexual violence had significantly lower PCS and MCS (PCS 42.2, SD = 5.3 and MCS 37.4, SD = 11.2) than women who had no history of sexual violence (PCS 51.0, SD = 7.5 and MCS 48.1, SD = 10.2, *p* < 0.001).
Chan OK (2010) [18]	Nausea and vomiting*	PW without symptoms of NVP: PCS: 67.92; MCS 68.36; PW with moderate symptoms: PCS: 56.93; MCS 60.86; PW with severe symptoms: PCS: 50.01; MCS: 50.23 The variations between the PCS and MCS of the 3 groups are all significant. (*P* < 0.01). All QOL dimensions are affected by NVP (*p* < 0.05)
Chang SR (2014) [39]	Pregnancy stage *, Experience of infertility *, Medically Assisted Reproduction *, Number of Pregnancies, Spontaneous Abortions, Parity *, Medical Condition *, Pregnancy wanted *	Factors associated with PCS: Pregnancy stage (beta = − 7.79, p < 0.001), experience of infertility (beta = − 6.39, *p* = 0.03). Factors associated with MCS: stage of pregnancy (beta = 3.31, p < 0.001), number of pregnancies (beta = − 7.12, *p* = 0.01), medical condition beta = − 4.08, *p* = 0.04). Factors associated with overall QOL: Pregnancy stage (beta = 1.64, p = 0.01), desired pregnancy (beta = 5.52, p = 0.04), medical condition (beta = − 5.29, p < 0.001).
Coban A. (2011) [35]	Back pain	No significant difference between PBP and NBP in the different areas of WHO-QOL-BREF: physical health *p* = 0.229; psychological health *p* = 0.069; Social relationship *p* = 0.125; Environment *p* = 0.790
Da Costa D (2009) [19]	Age* , Education *, Income*, Professional status* , Marital status * , Parity * , Weeks pregnant , Medical history* , Medical complications during pregnancy * , Anxiety related to pregnancy * Partner Support * , Social Support * , Sleep Problems * , Depressive symptoms * , Life Events *	Multivariable analysis: Sleep problems affected most QOL components (PF: b = − 0.17, *p* < 0.007; PR: b = − 0,19, *p* = 0.002; BP: b = − 0,35, *p* < 0.0001; GH: b = − 0.21, p < 0.0001; VT: b = − 0.25, p < 0.0001; SF: b = − 0.26, p < 0.0001, MH: b = − 0.20, p < 0.0001). A depressive mood was an independent determinant in 6/9 of the QOL dimensions (BP: b = − 0.13, p = 0,.039, GH: b = − 0.28, p < 0.0001, VT: b = − 0.40, *p* < 0.0001, SF = − 0.27, p < 0.0001; ER: b = − 0.50, *p* < 0.0001, MH: b = − 0.59, *p* < 0.0001), and anxiety also affected physical activity and limitations due to physical state. The life experience of the past year had a negative impact on physical activity, social functioning and mental health scores. Having complications during pregnancy affected physical activity and social functioning. Age, professional status, educational level and medical problems were linked to only one parameter of SF36.
Dall’Alba V (2015) [20]	Epigastralgia *, Gastroesophageal Reflux *	Epigastralgia: significant decrease in PR (*p* = 0.009) and of SF (*p* = 0.020); RGO: significant decrease in PR (*p* = 0.004) and of ER (*p* = 0.002)
De Pascalis L. (2012) [40]	Medically assisted fertility*	PW having a medically assisted pregnancy: PCS: 40.0 then 35.97; MCS: 52.32 then 53.02; PW having conceived spontaneously: PCS: 44.78; then 38.86. MCS: 51.08 then 53.65 Physical well-being scores significantly lower in the medically assisted pregnancy group than in the spontaneous conception group (*P* = 0.033) in the categories: physical health limitations, vitality, social functioning. Physical well-being scores of PW with medically assisted pregnancy decreased during pregnancy, more significantly than spontaneously conceived PW. (*P* = 0.008)
Elsenbruch S. (2006) [21]	Social support*	Low social support was statistically associated with a reduced QOL (For PCS: F = 11.53, *P* < 0.001, For MCS: F = 90.60, P < 0.001). On the contrary, the group where social support was high had better QOL. (P < 0.001)
Emmanuel EN (2012)	Age, Relationship status, Length of relationship, Level of education, Parity, Timing of first antenatal visit, Socialupport	Social support: beta = 0.21 [− 0.04,0.47] was not significant during pregnancy
Emmanuel EN (2014) [41]	Age *, Number of pregnancies *, Marital status *, Ethnicity *, Maternal stress *	PW between 25 and 29 years of age had a better RF, PR, BP than the others (PF: F = 11.07 *P* = 0.001; PR: F = 5,17 p = 0,006; BP: F = 11.01 *p* = 0.001) Caucasian and Asian PW had higher SF and MH scores than other ethnic groups (SF: F = 2.65, *p* = 0.02; MH: F = 2.42 *p* = 0.03) Single PW had higher SF and MH scores than other ethnic groups (SF: F = 2.65, p = 0.02; MH: F = 2.42 p = 0.03) A significant relationship is found between the different components of SF12 and maternal stress except for BP (PH *r* = − 3.87; PR *r* = − 7.79; BP: r = − 7.79; GH: *r* = − 6.63; VT = − 7.70; SF: *r* = − 12.61; ER: *r* = − 12.66; MH: r-12.80 *p* < 0.01)
Fatemeh A (2010) [32]	Age*, Gestational age*, Gravidity*,Education*, Wanted pregnancy*, life satisfaction*, Income	Age: < 25 years: GH = 63.68; PF = 64.71; SF = 66.37; MH = 68.33; > 25 years: GH = 60.44 p = 0.009; PF = 20.60 *p* = 0.010; SF = 61.47 p = 0.009; MH = 64.81 *p* = 0.018 Gestationnal age: < 20 weeks: PF = 65.51; BP = 56.89; > 20 weeks: PF = 60.72 *p* = 0.013; BP = 57.22 *p* = 0.022 Gravid 1 N: GH = 63.23; PF = 62.93; RP = 56.86; SF = 65.35; MH = 67.73; VT = 56.97; > 2 N: GH = 55.42 p = 0 .000; PF = 56.16 *p* = 0.011; RP = 52.09 *p* = 0.038; SF = 56.52 p = 0.002; MH = 60.09 *p* = 0.000; VT = 52.56 *p* = 0.048 Significant association between levels of education and PF and MH, between wanted pregnancy and RE and MH, life satisfaction and SF and MH (figures not available)
Gezginç K (2008) [34]	Obsessive Compulsive Disorder	Patients with OCD: Physical health: 49.92 +/− 15.44 Mental health: 46.20 +/− 15.98 Social relationships 44.96 +/− 15.00 Environment 50.32 +/− 9.88 PW control group: Physical health: 61.96 /− 10.08 and p = 0.002; Mental health: 66.32 +/− 10.47and *p* < 0.0001; Social relations: 67.12 +/− 11.92 and p < 0.0001; Environment 69.60 +/− 10.23 and p < 0.0001
Gharacheh M. (2015) [22]	Domestic violence*	6 SF36 sub-scales are lower for abused women than for non-abused women: PR (p = 0,041) GH (*p* = 0.003) PCS (p = 0.009) VT (p = 0.011) SF (*p* = 0.05) ER (*p* = 0.037) MH (*p* = 0.035) MCS (*p* = 0.07)
Haas JS (2004) [42]	Age, Ethnicity, Marital Status, Level of Education, Body Mass Index, Obesity * , Financial problems*, Physical exercise *, Depressive Symptoms *, Pre-conception smoking *, History of alcohol addiction *, Symptoms associated with pregnancy *, Medical background, Medical complications	Factors associated with poor health: financial problems (OR = 2.11, IC (1.49–2.98)), low physical function before pregnancy (OR = 1.99, IC (1.37–2.88)), depressive symptoms (OR = 2.30; IC (1.61–3.29)), obesity = (OR = 1.70; IC (1.16–2.48), lack of physical exercise, (OR = 1.12; IC (0.77–1.63) Smoking during the 3 months prior to conception (OR = 1.04; IC (0.65–1.68)), History of alcohol dependence (OR = 1.55, IC (1.00–2.39)) Indigestion was associated with poor physical function (OR = 1.49; IC (1.04–2.13)). Dizziness (OR = 2.06; IC(1.57–2.71), back pain (OR = 1.71; IC(1.27–2.31)), breathlessness (OR = 1.32; IC(1.02–1.71)) were associated with a low vitality score. Dizziness, indigestion, shortness of breath and sleep disorders were associated with depressive symptoms.
Hama K (2008) [51]	Number of pregnancy*	PF, RP, GH: no significant differences between nulliparous and multiparous women BP and VT: scores higher in multiparous in the 3rd and 4th monthes of pregnancy, and lower in the 9th month of pregnancy; SF: higher in multiparous from the 3th month to the 7th month and lower in the 9th month; RE and MH: scores higher in multiparous up to 6th month pregnancy (p < 0,05) (no figures avalaible)
Jommen J (2005) [23]	Depression*, Anxiety*.	The PW group with depression had significantly lower QOL scores for all SF36 parameters (*p* < 0.05) except for physical function (*p* = 0.73) and vitality (*p* = 0.09). The PW group with clinically significant anxiety levels had significantly lower QOL scores in terms of physical pain (*p* = 0.02) and general health (*p* < 0.001)
Lacasse A (2008) [24]	Nausea and Vomiting *, Ethnicity *, Age *, Medical Insurance, employment, Educational level, Income, Physical exercise *, Alcohol and tobacco *	PW without NVP: PCS = 49,5; MCS = 49; PW with NVP: PCS = 43; MCS = 46 *P* < 0.001 AND *P* = 0.003 PCS age: b = − 0.32 *p* = 0.006; Country of birth MCS: b = 3.02 *P* = 0.035; Hispanic ethnicity MCS: b = 4.88 p = 0.035 Physical exercise: PCS: b = 3.47 *p* = 0.001 MCS: b = 2.17 *p* = 0.031; Coffee: PCS: b = 2.84 p = 0.006; Alcohol:: PCS: b = 3.38 *p* = 0.045
Lau Y (2011) [25]	Perceived Stress *	Significant association between perceived stress and PCS (beta = − 0.501, p < 0.001) and MCS (b = − 0.115, p < 0.001)
Li J (2012) [26]	Depression*, Age*, Body Mass Index*, Educational Level* Physical exercise, History of smoking, History of alcohol abuse, Income*, Wanted pregnancy, Weeks pregnant*, Parity, History of abortion, Pregnancy complications	The PW group presenting depression had significantly lower QOL scores for all SF36 parameters (*p* < 0.002) PW not depressed: PCS = 49.13 (6.95); MCS = 48.67 (7.33)) (p < 0.001); PW depressed: PCS = 46.43 (7.42); MCS = 39.62 (7.94) (p < 0.001) Age: PCS *r* = − 0.170 (p < 0.001); MCS: *r* = 0.108 (*p* < 0.05); BMI: MCS: *r* = 0.114 (p < 0.05); Education level: MCS *r* = 0.203 (p < 0.001) Monthly income: MCS *r* = 0.183 (p < 0.001); Week of pregnancy: PCS: *r* = 0.145 (p < 0.001); MCS *r* = 0.118 (p < 0.05) Multivariable: PCS: EPDS: beta = − 0.232 (*p* < 0.0001); Age: beta = − 0.179 (p < 0.0001); WP: beta = − 0.129 (*p* = 0.004); Employment: beta = − 0.111 (*p* = 0.012); Marital status: beta = − 0.107 (*p* = 0.017); MCS: EPDS: beta = − 0.662 (p < 0.0001); BMI: beta = 0.129 (p < 0.0001); Wanted pregnancy: beta = 0.086 (*p* = 0.018)
Liu L (2013) [27]	Ethnicity	Black patients: PF = 58; PR = 52; BP = 69; GH = 71; VT = 47; SF = 72; ER = 63; MH = 79; White patients: PF = 77; PR = 78; BP = 83; GH = 83; VT = 58, SF = 87; ER = 89; MH = 83. Black women had significantly lower QOL scores in physical activity (*p* < 0.001), physical limitation (p < 0.001), physical pain (p = 0.02) (*P* = 0.01), social functioning (*p* = 0.002), limitations related to mental state (p < 0.001). After adjusting for depressive symptoms, social support and BMI, these differences become no longer statistically significant.
Mckee MD (2001) [28]	Depression* Social support*	Depression is strongly and negatively correlated with all subscales of SF 36 MH (r = − 0,69), VT (r = − 0,63), SF (*r* = − 0.62), and ER (*r* = − 0.54) Social support was related to MH (*r* = 0.24), ER (*r* = 0.19), and SF (r = 0.14).
Moyer CA (2009) [30]	Optimism*	Optimism is positively associated with MCS (*p* = 0.001), VT (*p* = 0.041), and MH (p < 0.001)
Nakamura Y (2012) [36]	Comfort*, Hospitalisation*	The sense of comfort and the 6 areas of HRQOL were significantly lower for inpatients than for ambulatory PW and non-pregnant women (*p* < 0.05 and p = 0.001). A significant correlation was found between subjective comfort and QOL in vitality (p < 0.001) and mental health (p < 0.001)
Nicholson WK (2006) [31]	Ethnicity*, Income*, Social Support* Depressive Symptoms*, Multi-parity* Chronic illness	Ethnicity: African Americans / Whites: PF: − 15 (− 22;-8) RP: − 28 (− 41, − 15), GH: − 11 (− 19; − 13)(, VT: − 13(− 28;-3): SF: − 7 (− 8; − 6), Income: GH -10 (− 17; − 3), SF -4 (− 5, − 3), MH -9 (− 15; − 4); Social support: PR: 30 (6,55) GH: 9 (5,15), MH: 14 (12; 16) Multi-parity (> 2 previous deliveries): PR: 18 (7;30), SF: 17 (16, 18); Depressive symptoms: PR: − 50 (p = 0.006); BP: − 12 (*p* = 0.01); GH: − 10 (p = 0.01); VT = − 20 (*p* > 0.001); SF = − 38 (*p* < 0.01); ER: − 40 (p < 0.01); MH = − 24 (p < 0.01); Still significant in multivariable analysis
Olsson C (2004) [32]	Back pain	The QOL was lower in the PW group with back pain group (16 ± 16) (*p* = 0.000), also found in the sleep subcategories (P = 0.003), energy (*p* = 0.024), pain (p = 0.000) and physical mobility (p = 0.000). The PW group with back pain had a higher rate of occupational withdrawal (57%, *p* = 0.005), aptitude for household work (62%, p = 0.002), Social life (35%, *p* = 0.007) and leisure time (68%, *P* = 0.001)
Ramirez-Vélez (2011) [34]	Age*, Educational Status*, Socioeconomic levels*, Work status*, Marital Status*, Gestational weeks	Age: PF: r = − 0.17 (p < 0.05); Educational Status: GH: *r* = 0.34 (p < 0.001) Socioeconomic levels: PR *r* = 0.17 (p < 0.05); GH *r* = 0.29 (p < 0.001); SF: r = 0.19 (p < 0.05); ER: *r* = 0.27 (p < 0.01); MH: *r* = 0.22 (p < 0.01) Occupation (housewife): PR: r = 0.20 (p < 0.01); GH: r = 0.19 (p < 0.05); Marital Status (Being married or cohabiting): PF: *r* = 0.15 (p < 0.05) PR: *r* = 0.16 (p < 0.05) GH: *r* = − 0.22 (p < 0.01) VT: r = 0.19 (p < 0.05) SF: *r* = − 0.23 (p < 0.01) ER: *r* = − 0.19 (p < 0.01) MH: *r* = − 0.25 (p < 0.01)
Setse R (2008) [43]	Depressive symptoms*	In the 1st trimester: PW not depressed: PF = 82; PR = 59; BP = 77; GH = 65; VT = 47; SF = 81; ER = 87; MH = 79 Depressed PW: PF = 78; PR = 39; BP = 59; GH = 51; VT = 35; SF = 54; RE = 41; MH = 57 Depressed PW had a significantly worse QOL on the following SF36 conditions: physical pain, vitality, social functioning, functioning related to mental health, mental health. These PW had QOL scores of 10–23 points and 19–31 points lower in the 2nd and 3rd trimester
Shishehgar S (2014) [33]	Perceived stress during pregnancy *	Significant relationship between QOL and stress rate (*p* = 0.026, *r* = 0.007)
Tavoli Z (2016)	Domestic violence*	Non-abused women: PF = 68.7; RP 43.2; BP = 70.7; GH = 69.5; VT = 55.6; SF = 70.5; RE = 46.0; MH = 63.5; Abused women: PF = 53.9 (p < 0.0001); RP = 25.8(p < 0.0001); BP = 64.2 (NS); GH = 61.4 (p < 0.01); VT = 43.5(p < 0.0001); SF = 55.8 (p < 0.0001); RE = 20.1 (p < 0.0001); MH = 55.3(*p* < 0.003); General Health: physical violence OR = 2.13 (*p* = 0.03), Mental Health: psychological violence OR = 1.24 (p = 0.04)
Tendais I (2011) [44]	Physical activity*	PW who had low physical activity before and ^1st^ trimester had better mental health at 19–24 WP than active PW who became less active 10–15 PW (*p* = 0.015).
Tsai SY (2016) [45]	Sleep patterns	Pittsburgh Sleep Quality Index: T1: MCS b = − 1.40 PCS b-1.07; T2: MCS b = − 0.74 PCS b = − 0.88; T3: MCS b = − 1.42 PCS b = − 0.68; *P* < 0.01
Vinturache A (2015) [47]	Medically assisted reproduction*, Maternal age Gravidity, Pre-pregnancy BMI*, Physical function before pregnancy*, Mental function before pregnancy	Prior to 25 WP, AC women (who had MAR) had better mental QOL (*p* > 0.05) but a lower physical health score (*p* = 0.031) than SC women (spontaneous conception). At 34–36 WP, one difference persisted for the physical symptoms between the 2 Groups (p < 0.05). No post-partum health-related QOL differences.
Wang P (2013) [48]	Employment *, Happy being pregnant *, Education *, Primipara *, Age	Employment: b = − 4.05, p < 0.001 for mental health; B = − 3.06 p = 0.002 for social health; B = − 3.39, p = 0.001 for general health; Happy being pregnant: b = 8.70, p = 0.01 in the mental health component and b = 6.94, p = 0.03 for general health; level of education b = − 2.10, p = 0.04 for the mental health component Primipara: b = 0.04, p = 0.04 for general health

*Significantly associated with the quality of life of pregnant women (*p* < 0.05). Abbreviations: *PW* Pregnant women, *QOL* Quality of life, *WP* Weeks of pregnant, *PCS* Physical Component Summary, *MCS* Mental Component Summary, *T1* First trimester, *T2* Second trimester, *T3* Third trimester, *PP* Postpartum, *PF* Physical Functioning, *PR* Physical role, *BP* Bodily Pain, *GH* General Health, *VT* Vitality, *SF* Social Functioning, *ER* Emotional role, *MH* Mental Health

#### Socio-demographic factors

The following socio-demographic factors were strongly associated with a better quality of life in 15 studies: mean maternal age, primiparity, early gestational age, the absence of economic problems, a high educational level, being employed, being married, having family and friends. With less consensus, belonging to an ethnic minority and alcohol consumption have also been associated in 2 studies with a poorer quality of life.

#### Physical factors

Medically assisted reproduction, obstetric complications, medical history, possible hospitalisation and obesity were factors frequently indicating a poor quality of life during pregnancy (in 9 studies). Physical symptoms associated with pregnancy such as nausea and vomiting, epigastralgia, reflux, shortness of breath, dizziness, back pain and sleep problems affected women’s quality of life. Exercise was a factor that improved the quality of life of pregnant women. With less consensus, we found other factors associated with a poorer QOL such as smoking during the months prior to conception, a history of alcohol dependence and poor comfort.

#### Psychological factors

Eight studies have shown that symptoms of depression, anxiety, and stress were factors that had a strong negative impact on the quality of life of pregnant women. Sexual and domestic violence was linked to a lower quality of life, as well as the experience of life-threatening events and the experience of infertility. Happiness at being pregnant and being optimistic were factors related to a better quality of life.

## Discussion

### Summary of results

Pregnant women, especially during the third trimester, had significantly lower quality of life scores than non-pregnant women of the same age. Physically, the quality of life decreased significantly during the course of the trimesters. On a psychological level, several studies reported an increase in quality of life relative to mental health during pregnancy, and in others psychological stability was seen. Many factors were associated with the quality of life in pregnant women. Some factors associated with higher well-being were socio-demographic (first-time pregnancy, a favourable socio-economic status, social support, partner support). Similarly, the desire to be pregnant and moderate physical activity were factors associated with a positive quality of life. A lesser quality of life was attributed to physical factors, (such as complications during pregnancy, medically assisted reproduction, obesity prior to conception, physical symptoms such as nausea and vomiting, sleep difficulties), and otherwise attributed to psychological factors, (such as anxiety and stress during pregnancy and depressive symptoms).

### Strengths and limitations of the study

To our knowledge, this is the only systematic review of international literature aimed at synthesizing data regarding the quality of life of pregnant women. Many factors have been studied, considering the different dimensions of the quality of life, for pregnant women in general good health. This work also has its limitations: the research only included articles written in English or French. Therefore, the issue of generalizability should be discussed. In addition, two other articles could not be read in their entirety. Within the selected studies, few studies were multi-centric and only one study used information from a large national survey. In addition, we found a high degree of heterogeneity in the methodology for population selection, in choosing the quality of life scale, as well as in the results presented (dimensions of SF-36, composite scores, etc) complicating the synthesis study. Specific HRQOL questionnaires were not included because they are focused more on specific problems in pregnancy (such as nausea and vomiting) rather than on women’s overall well-being and their quality of life. With regard to the quality of these studies, three studies did not explicitly take into account the confounding factors in the presentation of the results [22, 38, 47], which may lead to confusion bias. In addition, a large number of studies were cross-sectional. These studies did not establish an associative cause-and-effect relationship.

### Research and policy implications

In some countries, the quality of life of pregnant woman has been little studied. In France, for example, only one study provided information on pregnant women well-being [6] (study not included in our analysis – cf. exclusion criteria). In this study, the mental health of pregnant women was measured through the following question: “On a psychological level, how did you feel during your pregnancy? Well, Quite well, Quite poor, Poor”. Of the 14,326 women interviewed, 8.9% reported poor self-rated mental health during pregnancy. Moreover, sociodemographic characteristics indicative of social disadvantage were associated with a higher-risk of poor quality of life; sometimes, a social gradient was observed. [6, 52]. Since SF-36 is a generic scale, it allows comparisons with chronic pathologies such as diabetes. The results obtained concerning physical activity and pain in pregnant women can be compared to those obtained for chronic diseases such as cardiovascular diseases, diabetes and cancer [18]. A study by Sprangers et al. in 2000 found an average value of 58 in the “Physical Functions” and 64 in the “Bodily Pain” categories concerning diabetic patients [53]. In comparison, we found values between 53 and 77 for “Physical Functions” and between 48 and 74 for “Bodily Pain” in pregnant women. Moreover, quality of life refers to subjective elements that may vary from one culture to another. In the Coban et al. study undertaken in three countries over three continents: China, Ghana, USA [25], the subjective concepts of “well-being” or “vitality” were perceived and measured in very different ways on the three continents. This study did not focus on the quality of life of fathers. According to the *Abassi* et al. study, the quality of life of fathers was significantly higher during pregnancy and postpartum than that of their partner [39]. Specifically, their physical quality of life was significantly higher. Their mental quality of life was close, and there was no significant difference between them and their partner in two studies where this was studied [41, 43]. Finally, we believe that it is necessary to systematically screen women having a poor quality of life during pregnancy. Studies have to determine whether a single question is sufficient in clinical practice or whether it is preferable to have a more specific questionnaire before the follow-up consultation.

## Conclusions

Health-related quality of life refers to the subjective assessment of patients regarding the physical, mental and social dimensions of well-being. Women’s subjective perception of their health-related quality of life is an essential measure of the quality and effectiveness of maternal and child health interventions. However, few women (less than 20%) speak spontaneously about their psychological ill-health to a health professional. Health authorities’ recommendations are needed to better detect a poor quality of life of pregnant women and to evaluate the impact of care in terms of quality of life of pregnant women. Then, given the diversity of factors associated with the quality of life, the medical and paramedical professions need to work in cohesion with social agencies, networks, and associations.
